# Heterozygous OT‐I mice reveal that antigen‐specific CD8 ^+^ T cells shift from apoptotic to necrotic killers in the elderly

**DOI:** 10.1111/acel.13824

**Published:** 2023-03-22

**Authors:** Dorina Zöphel, Lea Kaschek, Romy Steiner, Sandra Janku, Hsin‐Fang Chang, Annette Lis

**Affiliations:** ^1^ Department of Biophysics, Center for Integrative Physiology and Molecular Medicine, School of Medicine Saarland University 66421 Homburg Germany; ^2^ Cellular Neurophysiology, Center for Integrative Physiology and Molecular Medicine, School of Medicine Saarland University Homburg Germany; ^3^ Present address: Department of Cardiac Surgery, Center for Biomedical Research Medical University of Vienna 1090 Vienna Austria

**Keywords:** adaptive immunity, aging, apoptosis, CD8^+^ T cells, cytotoxicity, necrosis, OT‐I, perforin

## Abstract

Numerous alterations in CD8^+^ T cells contribute to impaired immune responses in elderly individuals. However, the discrimination between cell‐intrinsic dysfunctions and microenvironmental changes is challenging. TCR transgenic OT‐I mice are utilized to investigate CD8^+^ T‐cell immunity, but their immunodeficient phenotype hampers their use especially in aging. Here, we demonstrate that using a heterozygous OT‐I model minimizes the current limitations and provides a valuable tool to assess antigen‐specific T‐cell responses even at old age. We analyzed phenotypic and functional characteristics of CD8^+^ T cells from OT‐I^+/+^ and OT‐I^+/−^ mice to prove the applicability of the heterozygous system. Our data reveal that OVA‐activated CD8^+^ T cells from adult OT‐I^+/−^ mice proliferate, differentiate, and exert cytolytic activity equally to their homozygous counterparts. Moreover, common age‐related alterations in CD8^+^ T cells, including naive T‐cell deterioration and decreased proliferative capacity, also occur in elderly OT‐I^+/−^ mice, indicating the wide range of applications for in vivo and in vitro aging studies. We used the OT‐I^+/−^ model to investigate cell‐intrinsic alterations affecting the cytotoxic behavior of aged CD8^+^ T cells after antigen‐specific in vitro activation. Time‐resolved analysis of antigen‐directed target cell lysis confirmed previous observations that the cytotoxic capacity of CD8^+^ T cells increases with age. Surprisingly, detailed single cell analysis revealed that transcriptional upregulation of perforin in aged CD8^+^ T cells shifts the mode of target cell death from granzyme‐mediated apoptosis to rapid induction of necrosis. This unexpected capability might be beneficial or detrimental for the aging host and requires detailed evaluation.

## Graphical abstract

A heterozygous OT‐I aging model reveals that the intrinsic cytotoxic capacity of antigen‐specific CD8^+^ T cells increases with age. High perforin expression in CD8^+^ T cells from elderly OT‐I mice results in target cells' membrane disruption, replacing granzyme‐mediated apoptosis by rapid necrosis induction. Our study further demonstrates that heterozygous OT‐I mice offer a widely applicable tool to assess age‐related alterations in antigen‐specific CD8^+^ Tcell response.

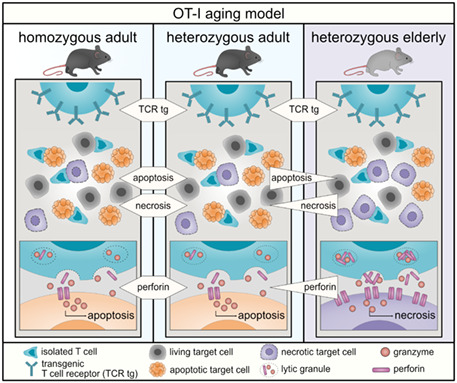

**Abbreviations**
APC: antigen presenting cell
CTL: cytotoxic T cell
FasL: Fas Ligand
OVA: ovalbumin
TCR: T cell receptor
TCR tg: transgenic T cell receptor

## 1 INTRODUCTION

Aging is accompanied by pronounced changes in immune cell functions, associated with an increased incidence of cancer, a higher risk for severe infectious diseases, and reduced responses to vaccination (Fulop et al., 2010; Ginaldi et al., 2001; Weinberger, 2021). Age‐related alterations affect almost all components of innate and adaptive immunity (Pinti et al., 2016). However, immunosenescence should be interpreted as a complex remodeling process rather than a progressive decline in immune function. Quantitative changes in cell subsets, altered cytokine production, and impaired receptor‐signaling modify the various interactions between immune cells, resulting in decreased or enhanced effector functions (Fulop et al., 2018; Goronzy et al., 2012).

Cytotoxic CD8^+^ T cells are substantially affected by these alterations, but the molecular mechanisms remain incompletely understood. Thymic involution and continuous antigen exposure result in reduced numbers of naive CD8^+^ T cells, leading to an inadequate immune response to novel pathogens (Cunha et al., 2020). The impaired expansion of antigen‐specific CD8^+^ T cells and decreased expression of effector molecules further contribute to inefficient pathogen clearance in elderly humans and mice (Jergović et al., 2019; Smithey et al., 2011). However, despite the overall reduced T‐cell response, we and others have revealed growing evidence that CD8^+^ T cells' intrinsic cytotoxic capacity increases with age (Saxena & Adler, 1999; Saxena et al., 1988; Zöphel et al., 2022). Separating cell‐intrinsic capabilities from microenvironmental changes may open new opportunities for developing and improving immunotherapeutic approaches. One of the major challenges is the heterogeneity of factors that shape the multifaceted process of immunosenescence. Aside from genetic predispositions, numerous extrinsic modulators such as nutrition, stress, and chronic viral infections have been identified, affecting immune competence with age on a very individual basis (Larbi et al., 2008). Although descriptive/correlative studies in elderly humans are essential to uncover age‐related deficiencies and their consequences for the organism, elucidating the underlying cellular mechanisms is often limited.

The use of mouse models has already provided comprehensive insights into the mechanisms of the aging process (Folgueras et al., 2018). The relatively short lifespan and genetic manipulation's feasibility overcome human aging research's inherent limitations. Studies performed in mice revealed numerous phenotypic and functional alterations in CD8^+^ T cells consistent with observations in elderly humans (Maue et al., 2009; Nikolich‐Žugich, 2014).

Model antigens such as ovalbumin (OVA) provide valuable tools for studying antigen‐specific immune responses. OT‐I transgenic mice express T‐cell receptors (TCR) on CD8^+^ T cells specific for the OVA_257–264_ peptide (SIINFEKL). They have become one of the most widely used TCR transgenic mouse models to investigate CD8^+^ T cells immunity in an antigen‐specific context. In aging studies, OT‐I T cells have mainly been used from young donor mice adoptively transferred into aged wild‐type recipients (Becklund et al., 2016; Decman et al., 2012; Li et al., 2012). Such cell transfer experiments reveal essential information about the influence of the aging environment on T cells' immune response but give little insight into cell‐intrinsic alterations occurring with age. To our knowledge, no data are currently available on how efficiently CD8^+^ T cells from elderly OT‐I mice can exert cytotoxic effector functions after encountering their specific antigen. Unfortunately, using a homozygous OT‐I model in aging research is challenging due to increased mortality and general health issues of these per se immunodeficient mice. To overcome this limitation, we established a heterozygous OT‐I aging model with extended life span. Here, we present a phenotypic and functional comparison of CD8^+^ T cells from homozygous and heterozygous OT‐I mice and reveal extensive changes in antigen‐specific cytotoxicity during aging.

## 2 RESULTS

### 2.1 Reduced incidence of splenomegaly in OT‐I^+/−^ mice

OT‐I mice carry a transgenic TCR on CD8^+^ T cells specific for the H‐2Kb‐restricted OVA_257‐264_ peptide, making them very attractive for studying antigen‐specific T‐cell responses in the context of aging. However, the inability to respond to other antigenic epitopes results in an immunodeficient phenotype frequently accompanied by the development of splenomegaly and markedly increased mortality, thus limiting the wide usage of OT‐I mice in aging research. Therefore, we generated a heterozygous aging model to improve the general health status by mating male OT‐I with female C57BL/6J mice.

Splenomegaly occurred only sporadically in adult heterozygous OT‐I mice (OT‐I^+/−^), and average spleen weight was decreased compared to their homozygous counterparts (Table 1). Higher spleen weights in female mice have previously been described in C57BL/6 mice (Menees et al., 2021) and also apply to OT‐I^+/+^ and OT‐I^+/−^. In addition, we observed a higher incidence of splenomegaly in female compared to male OT‐I mice.

**TABLE 1:** Body and spleen weight of OT‐I^+/+^ (*n* = 9–10) and OT‐I^+/−^ (*n* = 11–20) mice ± SEM.

	OT‐I ^+/+^ adult	OT‐I ^+/−^ adult	OT‐I ^+/−^ elderly
♂			
Body weight (g)	31.52 (±1.07)	30.36 (±0.38)	32.56 (±0.71)
Spleen weight (mg)	155.67 (±13.54)	107.23 (±5.05)	151.15 (±23.10)
**♀**			
Body weight (g)	22.29 (±0.53)	24.12 (±0.45)	26.25 (±0.42)
Spleen weight (mg)	179.10 (±19.64)	140.82 (±12.22)	166.07 (±23.76)

Spleen and body weight increases with age (Angenendt et al., 2020; Menees et al., 2021), and splenomegaly is a common finding in necropsies of elderly mice (Pettan‐Brewer et al., 2011). Accordingly, OT‐I^+/−^ mice showed increasing spleen and body weights during aging. Nevertheless, the average spleen weight of male and female elderly OT‐I^+/−^ mice was still lower than spleen weights of adult OT‐I^+/+^, respectively (Table 1).

By using a heterozygous OT‐I model, we were able to reduce the occurrence of splenomegaly and extend the life expectancy of the mice, allowing the characterization of antigen‐specific CD8^+^ T cells even in old age.

### 2.2 Preserved expression of transgenic T‐cell receptor in elderly OT‐I^+/−^ mice

The transgenic TCR expressed by OT‐I CD8^+^ T cells is derived from the OVA_257–264_‐specific CD8^+^ T‐cell clone 149.42 (Kelly et al., 1993) and consists of a rearranged V_α_2 and V_β_5 chain inherited via a single transgene (Hogquist et al., 1994). Since the presence of a functional TCR protein results in allelic exclusion and suppression of further endogenous TCR gene recombination (Sant'Angelo et al., 2001; Uematsu et al., 1988), one would assume that most CD8^+^ T cells from OT‐I^+/−^ mice express V_α_2/V_β_5 transgenic TCRs. Nonetheless, the distribution of CD8^+^ T cells carrying the OVA_257–264_‐specific TCR in OT‐I^+/−^ mice and the effect of aging on the maintenance of transgenic TCR expression have not been investigated so far. Besides, spontaneous partial loss of the V_α_2 or V_β_5 chain in OT‐I^+/+^ mice has been reported in some cases (Pritchard et al., 2016).

**FIGURE 1:**
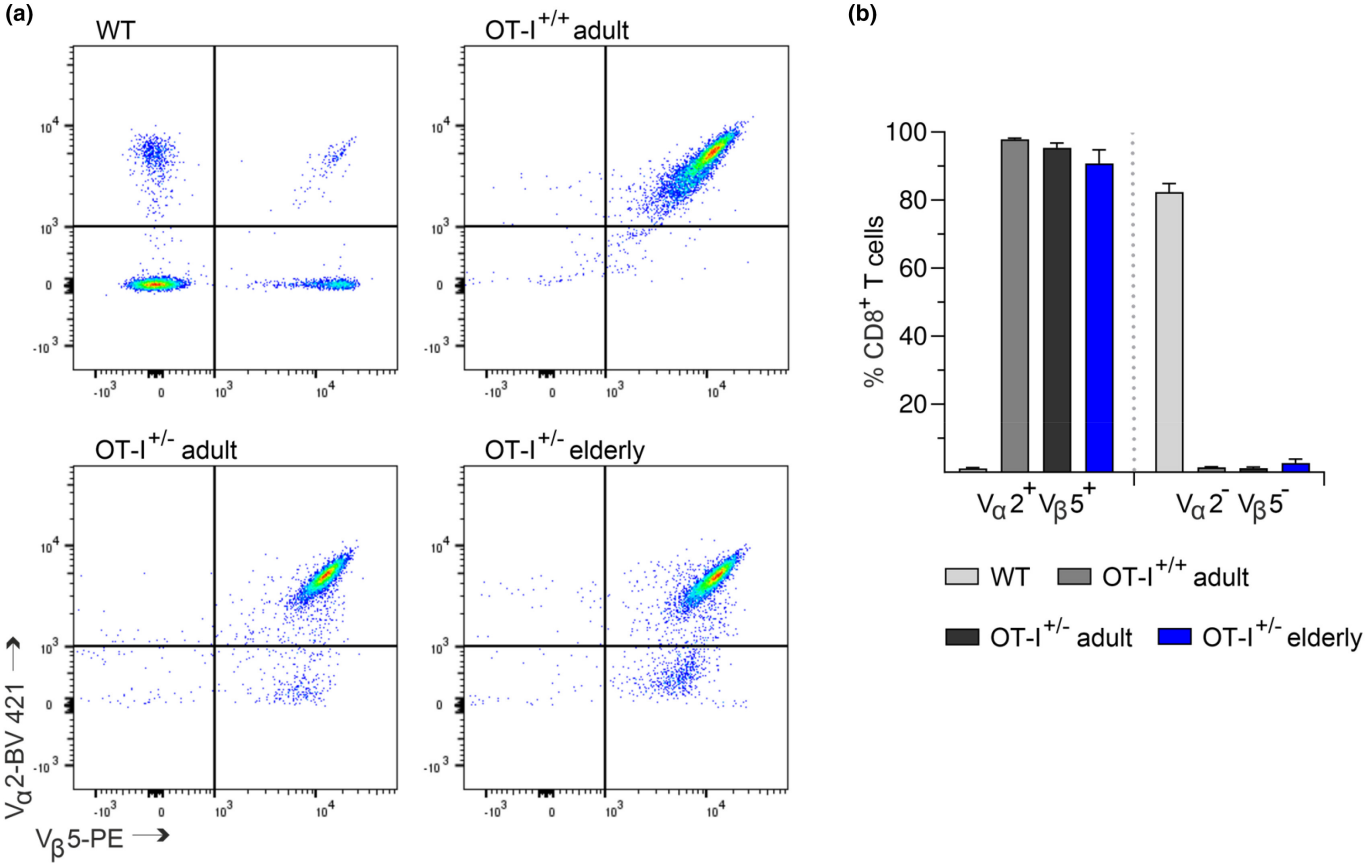
Conserved expression of the V_α_2/V_β_5 transgenic T‐cell receptor in CD8^+^ T cells from adult and elderly OT‐I^+/−^ mice. (a) Representative flow cytometric dot plots of V_α_2 and V_β_5 TCR chain expression in isolated CD8^+^ T cells from OT‐I^+/+^, OT‐I^+/−^, and C57BL/6J mice (WT). (b) Quantification of V_α_2^+^V_β_5^+^ and V_α_2^−^V_β_5^−^ cells among the CD8^+^ T‐cell populations. Data are presented as mean ± SEM, *n* = 4–5.

Therefore, we analyzed the expression of transgenic TCR by staining isolated CD8^+^ T cells from OT‐I^+/+^, OT‐I^+/−^, and C57BL/6J mice with anti‐V_α_2 and anti‐V_β_5 antibodies. Flow cytometry analysis revealed no significant differences between OT‐I^+/+^, OT‐I^+/−^, or the age of the mice. 98% of CD8^+^ T cells from OT‐I^+/+^ and 95% of CD8^+^ T cells from adult OT‐I^+/−^ mice carry the V_α_2/V_β_5 transgenic TCR (Figure 1a,b). In contrast, only 1% of CD8^+^ T cells from C57BL/6J mice express endogenously rearranged V_α_2 /V_β_5 TCRs.

In elderly OT‐I^+/−^ mice, 90% of CD8^+^ T cells are V_α_2^+^ V_β_5^+^, whereas 5% of CD8^+^ T cells express only the V_β_5 chain. Since up to 10% of CD8^+^ T cells from C57BL/6J mice use the V_β_5 chain for TCR gene recombination (Figure 1a), it remains unclear whether the 5% decrease in V_α_2^+^V_β_5^+^ TCR expression is caused by endogenous TCR rearrangements or if a partial loss of transgenic V_α_2 chain expression occurs more frequently during aging.

Taken together, most CD8^+^ T cells from OT‐I^+/−^ mice carry the OVA_257–264_‐specific TCR, whose surface expression is largely preserved during aging.

### 2.3 Age‐related alterations in subtype distribution and proliferative capacity of CD8^+^ T cells from OT‐I^+/−^ mice

A substantial shift of CD4/CD8 ratio towards CD8^+^ T cells is a well‐known characteristic of OT‐I mice (Clarke et al., 2000; Kaye et al., 1992). Furthermore, a reduced CD4/CD8 ratio is often associated with aging and reduced infection resistance.

**FIGURE 2:**
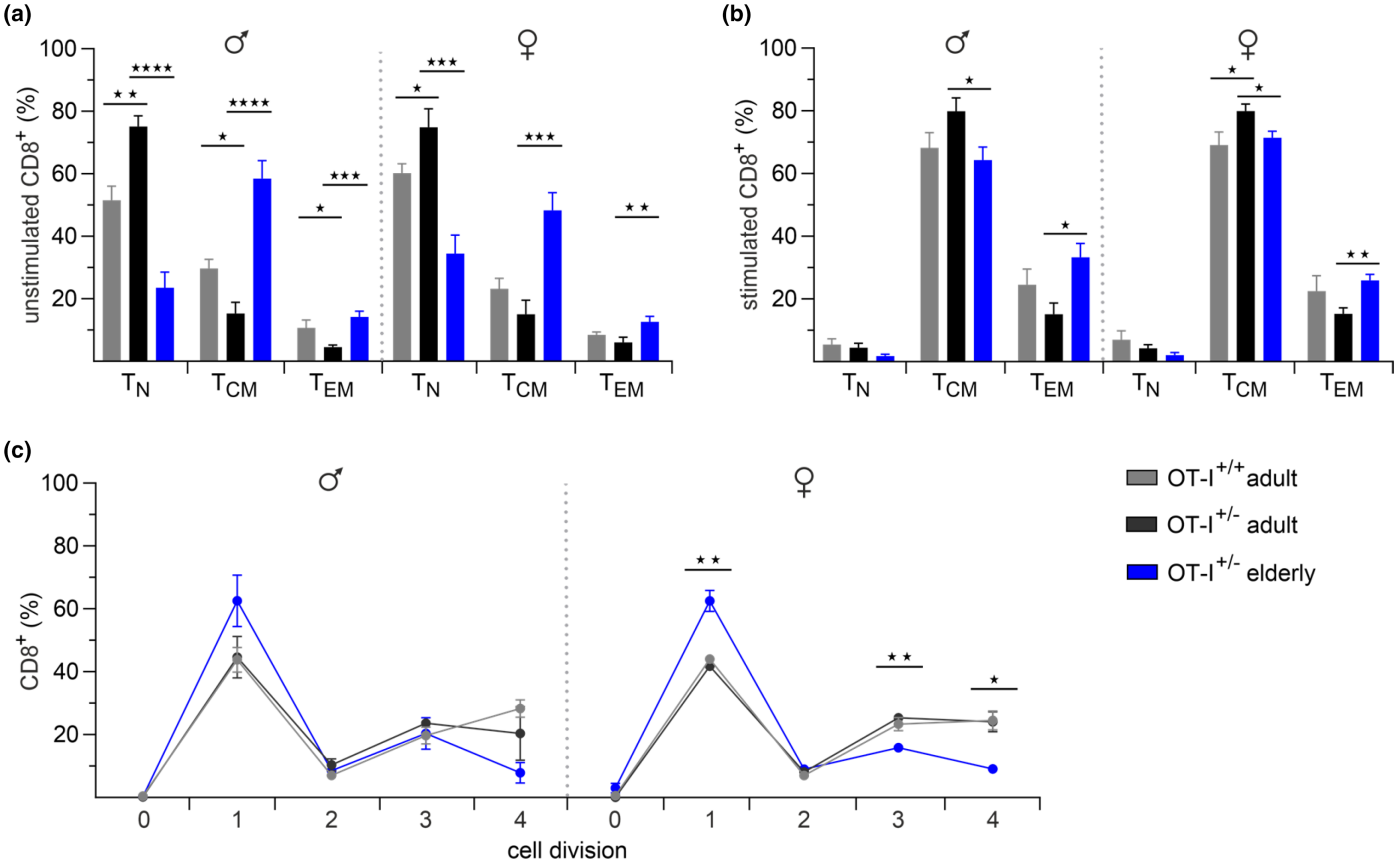
Subtype distribution and proliferative capacity of CD8^+^ T cells from OT‐I^+/+^ and OT‐I^+/−^ mice. Flow cytometry‐based analysis of subtype distribution in unstimulated (a) and stimulated (b) CD8^+^ T cells from male (*n* = 6–10) and female (*n* = 5–11) OT‐I^+/+^ and OT‐I^+/−^ mice. T‐cell subsets were defined based on CD62L and CD44 surface expression: T_N_: CD62L^high^CD44l^ow^, T_CM_: CD62L^high^CD44^high^, and T_EM_: CD62L^low^CD44^high^. (c) Flow cytometry‐based proliferation assay with CD8^+^ T cells from male and female OT‐I^+/+^ and OT‐I^+/−^ mice. CD8^+^ T cells were stimulated with irradiated E.G7‐OVA mouse lymphoma cells, and cell divisions were quantified through dilution of cytoplasmatic dye CFSE 48 h after stimulation. Data are presented as mean ± SEM, *n* = 3–5.

First, we analyzed the distribution of T‐cell subtypes in splenocytes from adult and elderly OT‐I^+/−^ mice compared to OT‐I^+/+^ (Figure S1, Figure 2a). Given the reported sex differences in T‐cell subpopulations and immune responses, not only during aging (Klein & Flanagan, 2016; Menees et al., 2021), we decided to characterize phenotypes of male and female mice separately. While the proportion of CD3^+^ T cells in splenocytes is comparable between OT‐I^+/+^ and OT‐I^+/−^ (Figure S1a), the predominance of CD8^+^ T cells is significantly more pronounced in adult OT‐I^+/−^ mice (Figure S1b). The CD8^+^ T cells’ population decreases with age, concurrently with increased percentages of CD4^+^ T cells in elderly male and female OT‐I^+/−^ mice (Figure S1b).

One hallmark of immunosenescence is the progressive decline in the naive T‐cell population (T_N_) along with the accumulation of memory T‐cell subsets, generally divided into central memory (T_CM_) and effector memory (T_EM_) T cells (Nikolich‐Zugich, 2008). We analyzed the distribution of T‐cell subsets in adult and elderly OT‐I mice by using the surface marker CD44 and CD62L to distinguish between T_N_ (CD62L^high^CD44^low^), T_CM_ (CD62L^high^CD44^high^), and T_EM_ (CD62L^low^CD44^high^) in CD8^+^ and CD4^+^ T cells. The majority of CD4^+^ T cells in all OT‐I cohorts show the phenotypic characteristics of T_EM_ (Figure S1c), whereas CD8^+^ T cells from adult OT‐I^+/+^ and OT‐I^+/−^ mice mainly consist of T_N_ (Figure 2a). Additionally, we observed a significantly higher proportion of naive CD8^+^ T cells in adult OT‐I^+/−^ mice compared to their homozygous counterparts. In elderly OT‐I^+/−^ mice, numbers of naive CD8^+^ T cells were significantly reduced, and T_CM_ are the predominant T‐cell population (Figure 2a).

Upon antigen encounter, naive T cells clonally expand, differentiate into effector T cells, and subsequently into memory T cells (Nikolich‐Zugich, 2008). A related shift in T‐cell subsets has recently been reported after in vitro polyclonal stimulation of CD8^+^ T cells, in which naive T cells differentiate primarily into T_CM_ and secondarily into T_EM_ (Angenendt et al., 2020; Zöphel et al., 2022).

To evaluate the OVA‐specific T‐cell response in adult and elderly OT‐I^+/−^ compared to OT‐I^+/+^ mice, we isolated CD8^+^ T cells from splenocytes and stimulated them with irradiated E.G7‐OVA mouse lymphoma cells, which constitutively express the H‐2Kb‐restricted OVA_257–264_ peptide. After 3 days of activation, the naive T cells’ population was greatly reduced and most CD8^+^ T cells were differentiated into T_CM_ in all OT‐I cohorts. Nonetheless, age‐related differences in subtype distribution remained significant after activation (Figure 2b).

Next, we assessed the proliferative capacity to confirm the appropriate activation of CD8^+^ T cells from OT‐I^+/−^ mice and to reveal possible changes in antigen‐specific T‐cell responses during aging. We labeled isolated splenocytes with CFSE and incubated them with irradiated E.G7‐OVA cells. Stimulated cells were then stained with anti‐CD8 antibody, and cell divisions were quantified by flow cytometry. 48 h after antigen encounter, virtually all CD8^+^ T cells divided at least once (Figure 2c). CD8^+^ T cells from adult OT‐I^+/−^ mice proliferated equally to OT‐I^+/+^ with the most significant proportion in division 1 (40%–45%) and several cells in divisions 3 and 4 (20%–25% in each). In elderly male and female OT‐I^+/−^ mice, we observed on average 20% more CD8^+^ T cells in division 1 and fewer cells in divisions 3 and 4, indicating a decline in cell‐intrinsic proliferative capacity with age (Figure 2c).

In summary, CD8^+^ T cells from adult OT‐I^+/+^ and OT‐I^+/−^ mice are phenotypically similar and respond equally to OVA‐specific TCR stimulation. Commonly described age‐related alterations in CD8^+^ T cells, like naive T‐cell deterioration, accumulation of memory T cells, and reduced proliferative capacity also occur in elderly OT‐I^+/−^ mice. Therefore, the OT‐I^+/−^ model provides a widely applicable tool for studying antigen‐specific T‐cell responses in the context of aging.

### 2.4 Enhanced cytotoxicity of OVA‐specific CD8^+^ T cells from elderly OT‐I^+/−^ mice

Despite the evidence that CD8^+^ T cells' immune response is significantly impaired with age, remarkably little is known about cell‐intrinsic alterations affecting their primary function, the cytotoxicity against infected or malignant target cells. We have recently reported the increased killing efficiency of cytotoxic CD8^+^ T cells (CTLs) from elderly C57BL/6J mice after polyclonal stimulation (Zöphel et al., 2022). Since physiological TCR activation is more complex, we decided to investigate whether this unsuspected phenotype also applies to CD8^+^ T cells from elderly OT‐I^+/−^ mice after encountering their specific antigen.

**FIGURE 3:**
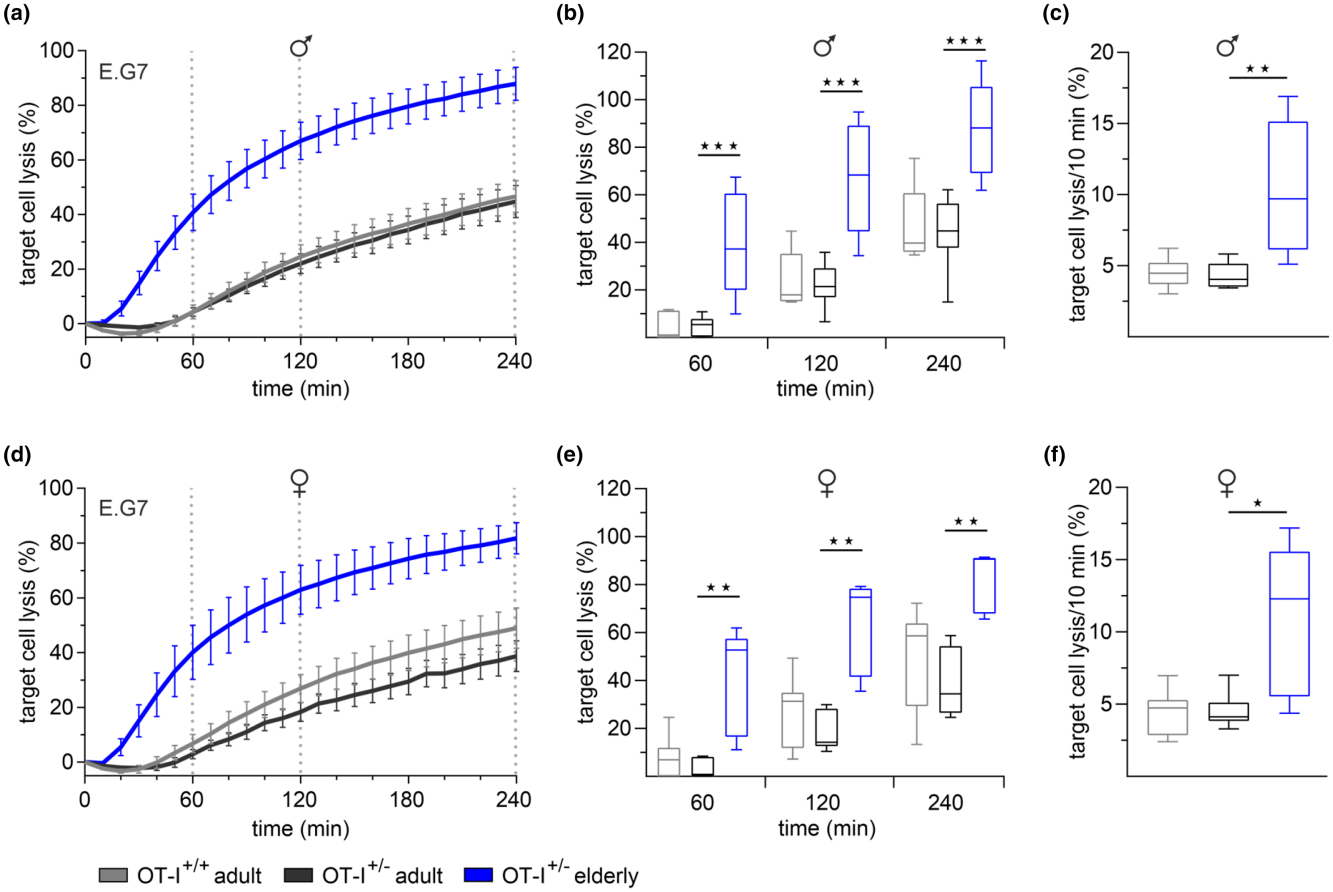
Faster cytotoxicity of CD8^+^ T cells from elderly OT‐I^+/−^ mice. Time‐resolved killing assays with CD8^+^ T cells from male (a) and female (d) OT‐I^+/+^ and OT‐I^+/−^ mice 3 days after stimulation with irradiated E.G7‐OVA mouse lymphoma cell line. E.G7‐OVA cells were used as target cells in an effector‐to‐target ratio of 10:1. Box plots represent the average target cell lysis after 60, 120, and 240 min (b, e) and the maximum target lysis per 10 min (c, f) as a measure of the kinetics. Data are presented as mean ± SEM, *n* = 5–11.

We quantified the cytotoxic activity using a real‐time killing assay, which allows a much more detailed characterization of cytotoxic behavior than conventional endpoint assays. We loaded E.G7‐OVA cells with the fluorescent dye calcein‐AM and kinetically monitored target cell death upon contact with activated OVA‐specific CD8^+^ T cells from adult and elderly OT‐I mice (Figure 3).

CTLs from adult OT‐I^+/+^ and OT‐I^+/−^ mice displayed 40%–45% target cell lysis after 240 min without significant differences in killing kinetics (Figure 3a,d). Consistent with previous observations (Zöphel et al., 2022), CTLs from all adult cohorts revealed delayed killing activity with less than 5% lysis within the first 60 min after target cell contact. In contrast, CTLs from elderly OT‐I^+/−^ mice showed significantly faster and more efficient cytotoxicity, with 40% target cells lysis after 60 min and 80% at the endpoint (Figure 3a,b,d,e). The quantification of the maximum target cell lysis per 10‐min interval, as a measure of the kinetics, indicated a 2.5‐fold higher maximum lysis rate of CTLs from elderly compared to adult mice (Figure 3c,f).

Our results support the evidence that enhanced CTLs’ killing efficiency with age is driven by cell‐intrinsic alterations, independently of the mode of TCR stimulation.

### 2.5 Age‐related alterations in mRNA expression of perforin, granzyme B and FasL in CD8^+^ T cells from elderly OT‐I^+/−^ mice

**FIGURE 4:**
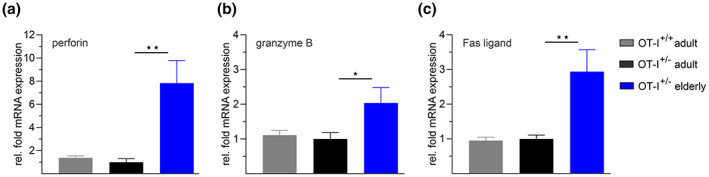
Increased mRNA expression of perforin, granzyme B, and Fas ligand in CD8^+^ T cells from elderly OT‐I^+/−^ mice. Normalized mRNA expression of perforin (a), granzyme B (b) and Fas ligand (c) in CD8^+^ T cells from OT‐I^+/+^ and OT‐I^+/−^ mice. Expression levels were normalized to the reference genes hypoxanthine‐phosphoribosyl transferase 1 (HPRT1) and TATA box binding protein (TBP). Data from adult OT‐I^+/+^ and elderly OT‐I^+/−^ mice are presented as relative fold change to the mRNA levels from adult OT‐I^+/−^ mice, respectively. Data are presented as mean ± SEM, *n* = 6–8.

An age‐related increased expression of the killing process's key components perforin, granzyme, and Fas Ligand (FasL) in CD8^+^ T cells has already been reported (Aggarwal & Gupta, 1998; Zöphel et al., 2022) and represents a plausible mechanism for enhanced cytotoxicity with age. To verify this conclusion, we analyzed the mRNA expression of perforin, granzyme B, and FasL in activated OVA‐specific CD8^+^ T cells from adult and elderly OT‐I mice. Expression levels from adult OT‐I^+/+^ and elderly OT‐I^+/−^ mice were normalized to reference genes and evaluated as relative fold change to normalized mRNA levels of CD8^+^ T cells from adult OT‐I^+/−^ mice (Figure 4). While mRNA expression of all three genes was comparable in adult OT‐I^+/+^ and OT‐I^+/−^ mice, we observed a significant twofold to threefold increase in FasL and granzyme B and a ninefold increase in perforin mRNA in CD8^+^ T cells from elderly mice (Figure 4a–c).

Since post‐transcriptional modifications can strongly influence the abundance of mRNA transcripts, we wondered whether increasing mRNA levels with age result from upregulated gene transcription or altered mRNA degradation. Therefore, we analyzed the mRNA decay of perforin, granzyme B, and FasL after treating activated CD8^+^ T cells from adult and elderly mice with the transcription inhibitor actinomycin D (Figure S2). 9 h after transcriptional inhibition, mRNA levels were substantially reduced for all three genes of interest. However, we could not detect significant differences in the kinetics of mRNA degradation between both age groups (Figure S2a–c). These data suggest that CD8^+^ T cells from elderly mice possess enhanced capabilities to transcriptionally upregulate the expression of genes involved in the cytotoxic pathways.

### 2.6 CD8^+^ T cells from elderly OT‐I^+/−^ mice kill their targets predominantly through rapid necrotic cell death

The rapid target cell death induced by CTLs from elderly mice leads to the assumption that the fast‐acting exocytosis pathway, rather than receptor‐mediated killing, decisively contributes to enhanced cytotoxicity with age. Perforin is essential for granzyme entry, which triggers cell death through caspase‐dependent apoptosis. In addition, high perforin concentrations can disrupt the membrane integrity of target cells resulting in necrotic cell death (Backes et al., 2018).

We analyzed the mode of target cell death in more detail using the FRET‐based apoptosis reporter pCasper (Shcherbo et al., 2009), which consists of a TagGFP and TagRFP fluorophore fused by a linker containing the caspase recognition sequence DEVD. The sensor was stably transfected into E.G7‐OVA cells and enables the discrimination between viable, apoptotic, primary necrotic (fluorescence loss without prior caspase activity) and secondary necrotic (fluorescence loss following initial apoptosis) target cells (Knörck et al., 2022). In our experimental settings, we could not observe any primary necrosis in adult and less than 10% in the elderly OT‐I CTLs. Therefore, we defined the sudden fluorescence loss accompanied by the typical morphological changes as necrotic cell death only.

**FIGURE 5:**
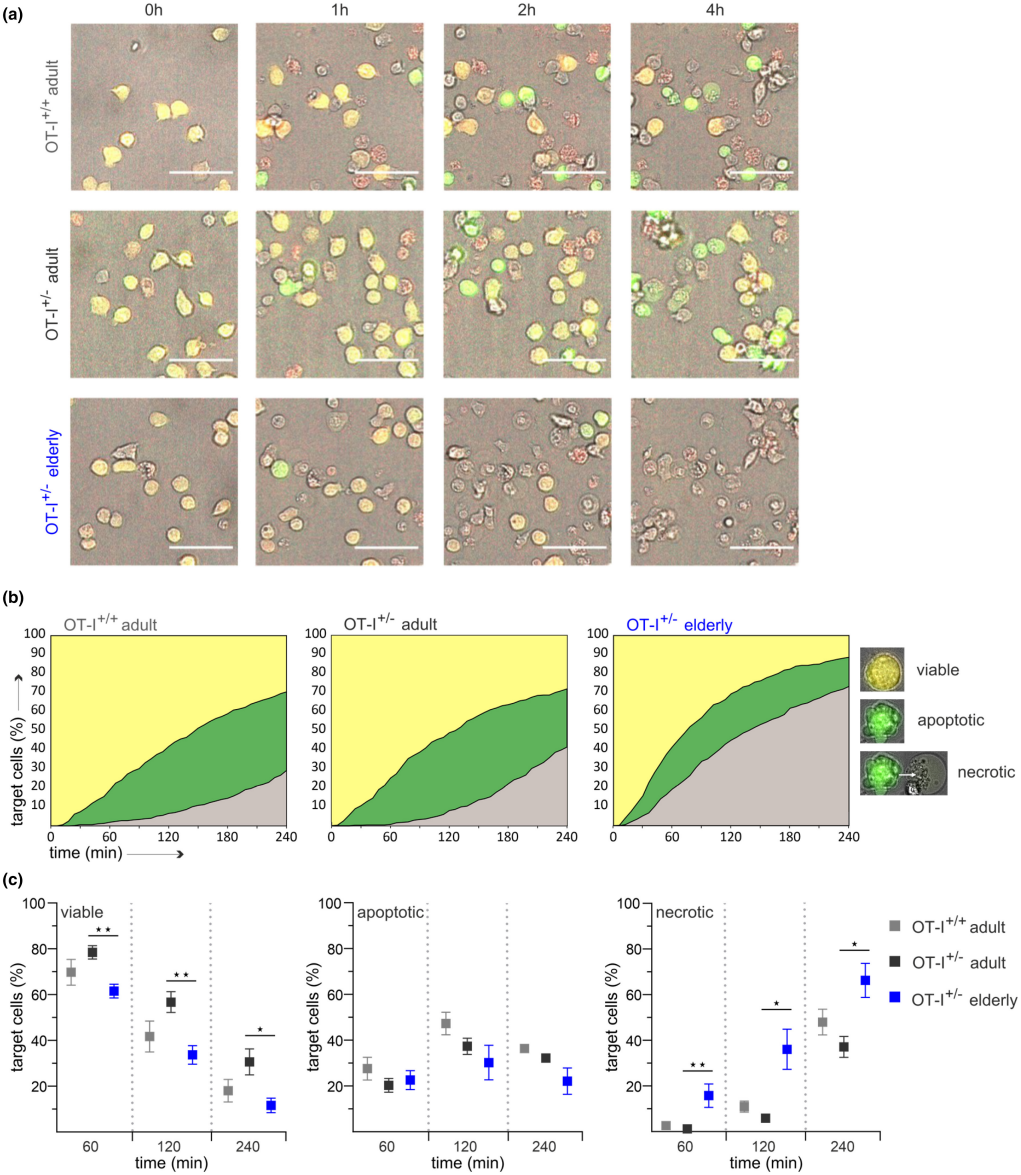
CD8^+^ T cells from elderly OT‐I^+/−^ mice induce rapid necrotic target cell death. Cytotoxicity assays with activated OVA‐specific CD8^+^ T cells from OT‐I^+/+^ and OT‐I^+/−^ mice. EG.7‐OVA pCasper cells were used as target cells to distinguish between viable (orange), apoptotic (green) and necrotic (fluorescence loss) cells with an effector‐to‐target ratio of 2:1. (a) Representative overlays of brightfield, GFP (green), and FRET (red) fluorescence at the indicated time points after effector cell contact. Images were acquired every 2 min for 4 h. (b) The percentages of viable, apoptotic, and necrotic cells were determined for each time point and plotted as color‐coded graphs over time. (c) Statistical quantification was performed at 60, 120, and 240 min. Data are presented as mean ± SEM, *n* = 4–6, scale bar 50 μm.

By using a high‐content imaging system, we kinetically monitored apoptotic/necrotic target cell death after contact with OVA‐specific CD8^+^ T cells from adult and elderly OT‐I mice (Figure 5a,b). As expected, the percentage of viable target cells was significantly lower with CTLs from elderly mice compared to their counterparts from the adults (Figure 5c). The analysis of target cell death induced by CTLs from adult mice revealed up to 50% apoptotic cells within the first 2 h, while the numbers of necrotic cells detected at this time were marginal. In contrast, CTLs from elderly mice induced rapid necrosis within minutes after target cell contact (Figure 5b). Moreover, the proportion of necrotic cells was significantly higher with CTLs from elderly mice at all analyzed time points, and in contrast to the adults, necrosis was the predominant form of cell death (Figure 5b,c).

We conclude that high perforin levels in CTLs from elderly mice shift the mode of target cell death from granzyme‐mediated apoptosis to rapid induction of necrosis caused by extensive membrane rupture.

## 3 DISCUSSION

Immunosenescence is a multifactorial phenomenon which inevitably occurs with age and substantially affects the susceptibility to cancer and infectious diseases in elderly individuals.

The shift to less naive and more memory T cells is probably the most noticeable alteration that weakens adaptive immunity during aging. On the contrary, lifelong antigen exposure in combination with compensatory mechanisms, particularly of the innate immune system, results in chronic inflammatory processes known as inflame‐aging (Fulop et al., 2018; Santoro et al., 2021). The growing understanding of this dynamic relationship favors a model in which aging is not fundamentally associated with the deterioration of immune cells’ effector functions. Considering immunosenescence as an adaptive remodeling process broadens the focus from solely elucidating functional deficits to unmasking retained intrinsic capabilities (Fulop et al., 2018; Jergović et al., 2019; Santoro et al., 2021). OVA‐specific CD8^+^ T cells from OT‐I mice are a well‐established model to investigate antigen‐specific T‐cell responses and have widely been used to study infectious diseases, autoimmunity, and cancer (Jenkins et al., 2006; Miyagawa et al., 2010; Rosato et al., 2019). Although predestined for studying age‐related T‐cell alterations, the immunodeficient OT‐I mice generally do not reach such a high age, thus receiving limited attention in aging research. In the present study, we demonstrate that using a heterozygous OT‐I model improves their health status and offers a valuable tool to characterize antigen‐specific CD8^+^ T cells even in old age. We proved the applicability of OT‐I^+/−^ mice by comparing phenotypic and functional characteristics of CD8^+^ T cells from adult homozygous and heterozygous mice. CD8^+^ T cells from adult OT‐I^+/−^ mice hardly differ in their transgenic TCR expression and show equal proliferation and cytotoxicity upon OVA‐specific activation. Therefore, we conclude that these mice are suitable for studying T cell‐mediated immunity. Besides, elderly OT‐I^+/−^ mice exhibit typical age‐related alterations in CD8^+^ T cells, including accumulation of memory T cells and decreased proliferative ability, reinforcing the vast field of new possibilities for using OT‐I^+/−^ mice as in vitro and in vivo aging models. The observed functional characteristics of CD8^+^ T cells show comparable tendencies between male and female mice, with minor differences in significance analysis. Whether the advantages of heterozygosity can be transferred to other TCR tg models highly depends on the underlying strategy and requirements of the study and requires individual investigations.

The OT‐I^+/−^ model has enabled us for the first time to assess the cytotoxic behavior of aged CD8^+^ T cells after antigen‐specific in vitro activation. It is commonly assumed that impaired T‐cell immunity is accompanied by reduced cytotoxicity, but remarkably, our knowledge of CD8^+^ T cells' intrinsic cytolytic capacity during aging is rather incomplete. We have recently reported the faster lysis kinetics of CTLs from elderly C57BL/6J mice after polyclonal stimulation with anti‐CD3/CD28 beads (Zöphel et al., 2022). Increased cytotoxicity with age has also been observed in concanavalin A‐activated splenocytes (Saxena & Adler, 1999; Saxena et al., 1988). Unfortunately, up to now, killing efficiency with higher age was not tested in an antigen‐dependent manner, which is the physiologically relevant stimulation. Our new data close this important gap: Antigen‐dependent cytotoxicity is faster with increased age. Thus, enhanced killing efficiency of aged CTLs is a generic intrinsic ability, independently of the mode of TCR stimulation.

We have previously shown that the enhanced cytolytic capacity is not due to the altered distribution of naive and memory T cells but to the ability of aged CD8^+^ T cells to upregulate perforin and granzymes (Zöphel et al., 2022). Elevated levels of perforin and granzyme in aged OT‐I CD8^+^ T cells support this. Interestingly, an age‐related increased expression of granzyme, perforin, and FasL has also been reported in human CD8^+^ T cells (Aggarwal & Gupta, 1998; Westmeier et al., 2020), suggesting that upregulation of genes involved in the cytotoxic pathway is a conserved mechanism occurring with age. Although the origin remains to be elucidated, these transcriptional modifications allow the assumption that increased cytolytic capacity is part of an adaptive process, counterbalancing other deficits in T‐cell immunity. However, the benefit for the immune response in the inflammatory environment is unclear.

Analysis of the mode of cell death of single cancer cells by CTLs from adult and elderly OT‐I mice revealed that, in contrast to the adults, CTLs from elderly OT‐I^+/−^ mice kill their targets predominantly through necrotic cell death. These results indicate that high perforin levels in aged CTLs cause extensive membrane disruption on target cells, replacing granzyme‐mediated apoptosis by rapid necrosis induction.

Necrotic cell death triggers a strong pro‐inflammatory immune response, often associated with tissue injury and the pathogenesis of diseases (Wallach et al., 2014). However, within the tumor microenvironment, we encounter a higher level of complexity. Cancer cell necrosis, as a result of hypoxia and metabolic stress in early stages, is a known characteristic of solid tumors (Hangai et al., 2021; Liu & Jiao, 2020), but its role in tumor progression is still not fully understood. Recent studies indicate that the release of intracellular components modulates the immune response in different ways, either suppressing or promoting tumorigenesis (Hangai et al., 2021; Liu & Jiao, 2020). How necrotic cancer cell death induced by infiltrating CD8^+^ T cells shapes the microenvironment remains elusive, but affecting the anti‐tumor immunity appears very likely.

In summary, antigen‐specific CD8^+^ T cells from elderly mice are very efficient and, interestingly, shift the mode of target cell death towards necrosis. This ability might be a curse or a blessing for the aging host and is worth considering in immunomodulatory approaches.

## 4 EXPERIMENTAL PROCEDURES

### 4.1 Mice

C57BL6/J and OT‐I TCR‐Tg mice (C57BL/6‐Tg (TcraTcrb) 1100Mjb/J) were purchased from Charles River Laboratories and bred in our own colonies. Heterozygous F1 mice (OT‐I ^+/−^) were bred in‐house from stocks of female C57BL6/J and male OT‐I TCR‐Tg mice. All animal experiments were approved by local authorities and performed in compliance with the German Animal Protection Law (Tierschutzgesetz, §11, Abs.1 Nr.1 and §8). Male and female mice between 12 and 24 weeks (adult) and 70 and 100 weeks (elderly) were used for experiments. Mice were housed under specific pathogen‐free (SPF) conditions and sacrificed by cervical dislocation at the designated time. Mice with splenomegaly or macroscopically visible tumors were excluded. Spleens were removed, and splenocytes were isolated with a 40 μm cell strainer (Corning®). Erythrocytes were depleted by incubation with a hypoosmolar solution. CD8^+^ T cells were negatively isolated using the Dynabeads™ Untouched™ Mouse CD8 Cells Kit (ThermoFisher).

### 4.2 Cell culture

OT‐I CD8^+^ T cells were stimulated with irradiated E.G7‐OVA mouse lymphoma cell line (a gift from the Institut de Recherche et d'Innovation Biomédicale, U1234, Université de Rouen Normandie) with a 1:2 CTL to APC ratio and cultured in AIM V™ medium (ThermoFisher), supplemented with 10% FCS, 100 U/mL recombinant human IL‐2 (Miltenyi), and 50 μM β‐mercaptoethanol. OT‐I splenocytes were stimulated in a 1:1 cell ratio, respectively. E.G7 cells were maintained in RPMI 1640, supplemented with 10% FCS, 1% penicillin/streptomycin, and 0.4 mg/mL G418. For live‐cell imaging experiments, E.G7 cells were stably transfected with pCasper‐pMax plasmid as previously described (Zhu et al., 2021) and cultured in RPMI 1640, supplemented with 10% FCS, 1% penicillin/streptomycin, 0.4 mg/mL G418, and 4 μg/mL puromycin.

### 4.3 Flow cytometry

All antibodies used for flow cytometry were purchased from Biolegend. For analysis of V_α_2 and V_β_5 surface expression, unstimulated CD8^+^ T cells were stained with FITC‐conjugated anti‐CD8, BV421‐conjugated anti‐V_α_2, and PE‐conjugated anti‐V_β_5 antibody. To evaluate subtype distribution, splenocytes and stimulated CD8^+^ T cells were stained with PerCP‐conjugated anti‐CD3, Pacific Blue™‐conjugated anti‐CD4, FITC‐conjugated anti‐CD8, PE‐conjugated anti‐CD44, and APC‐conjugated anti‐CD62L antibody. 2 × 10^4^ cells per sample were acquired on a BD FACSVerse™ flow cytometer (BD Biosciences) and analyzed using FlowJo version 10 (FlowJo, LLC).

### 4.4 CFSE proliferation assay

The proliferation of OT‐1 CD8^+^ T cells was analyzed using the CellTrace™ CFSE Cell Proliferation Kit (ThermoFisher) according to the manufacturer's instructions. CFSE‐labeled splenocytes were stimulated with irradiated E.G7‐OVA cells at a 1:1 cell ratio, as described above. For flow cytometric analysis, cells were stained with PerCP‐conjugated anti‐CD8 antibody. Cell division of stimulated CD8^+^ T cells was quantified after 24 h and 48 h using FlowJo version 10 (FlowJo, LLC).

### 4.5 Real‐time killing assay

Real‐time killing assays were carried out as previously described (Kummerow et al., 2014). E.G7 target cells were loaded with 500 nM calcein‐AM in AIM V™ medium containing 10 mM HEPES for 15 min at room temperature. Cells were washed once and settled into black 96‐well plates with clear‐bottom (Corning®) at a density of 2.5 × 10^4^ cells/well. OT‐I CD8^+^ T cells were gently added onto target cells at an effector‐to‐target ratio of 10:1. Target cell lysis was measured with a GENios Pro plate reader (Tecan) every 10 min for 4 h at 37°C using bottom reading mode.

### 4.6 Live‐cell imaging

E.G7‐pCasper cells were resuspended in AIM V™ medium without phenol red and settled into black 96‐well plates with clear‐bottom (PerkinElmer®) at a density of 2.5 × 10^4^ cells/well. OT‐I CD8^+^ T cells were added onto target cells at an effector to target ratio of 2:1. Images were acquired every 2 min for 4 h at 37°C and 5% CO_2_, using the high‐content imaging system ImageXpress Micro XLS (Molecular Devices). Target cell death was analyzed with ImageJ as previously described (Backes et al., 2018).

### 4.7 Quantitative real‐time PCR

Total RNA from stimulated OT‐I CD8^+^ T cells was isolated using TRIzol® Reagent (ThermoFisher). 0.8 μg of total RNA was reverse transcribed, and 1 μL of cDNA was used for real‐time PCR. Quantitative real‐time PCR assays were carried out in a CFX96™ Real‐Time System C1000™ Thermal Cycler (BioRad) using the QuantiTect SYBR Green PCR Kit according to the manufacturer's instructions (Qiagen). The expression of target genes was normalized to the expression of the reference genes HPRT1 and TBP. Relative expression levels were calculated using the ΔCq method (2^−ΔCq^).

To determine mRNA decay, stimulated CD8^+^ T cells were incubated with the transcription inhibitor actinomycin D at a final concentration of 10 μg/mL. After 0, 3, 6, and 9 h, total RNA was isolated, and qPCRs were performed as described above. Ct values of each time point were normalized to Ct values of *t* = 0.

QuantiTect primers:

**Table:** 

Target gene	Product	Cat. No.
Perforin	Mm_Prf1_1_SG	QT00282002
Granzyme B	Mm_Gzmb_1_SG	QT00114590
FasL	Mm_Tnfsf6_1_SG	QT00104125
HPRT1	Mm_Hprt_1_SG	QT00166768
TBP	Mm_Tbp_1_SG	QT00198443

### 4.8 Statistical analysis

Data are presented as mean ± SEM (*n* = number of experiments) if not stated otherwise. Data were analyzed using GraphPad (Prism) software version 8 and Microsoft Excel 2016. If Gaussian distribution was confirmed, unpaired Student's *t* tests were performed to evaluate statistical significance. If no Gaussian distribution was given, nonparametric Mann–Whitney tests were performed. Degrees of significance were set at *p < 0.05, **p < 0.01, ***p < 0.001 and ****p < 0.0001.

## AUTHOR CONTRIBUTIONS

A.L. and D.Z. designed the study. A.L., D.Z., and H.‐F.C. discussed and interpreted all data, and wrote the manuscript with input from all authors. D.Z. performed flow cytometry analysis and cytotoxicity assays. L.K. performed single target cell death experiments and analysis. R.S. established cell culture conditions for OT‐I CD8^+^ T cells and did preliminary experiments. S.J. performed all qPCRs. D.Z. performed statistical analysis and designed final figure layout.

## CONFLICT OF INTEREST STATEMENT

The authors declare that they have no competing interests.

## Supporting information


Figure S1–S2

## Data Availability

The data that support the findings of this study are available from the corresponding author upon reasonable request.
