# Supplementary material for: Heterozygous OT‐I mice reveal that antigen‐specific CD8 + T cells shift from apoptotic to necrotic killers in the elderly
Source: Aging Cell. 2023 Mar 22;22(6):e13824. doi: 10.1111/acel.13824 (PMC10265152; doi:10.1111/acel.13824)
Supplement: Supplementary file 1 — Figure S1–S2 [file ACEL-22-e13824-s001.docx]

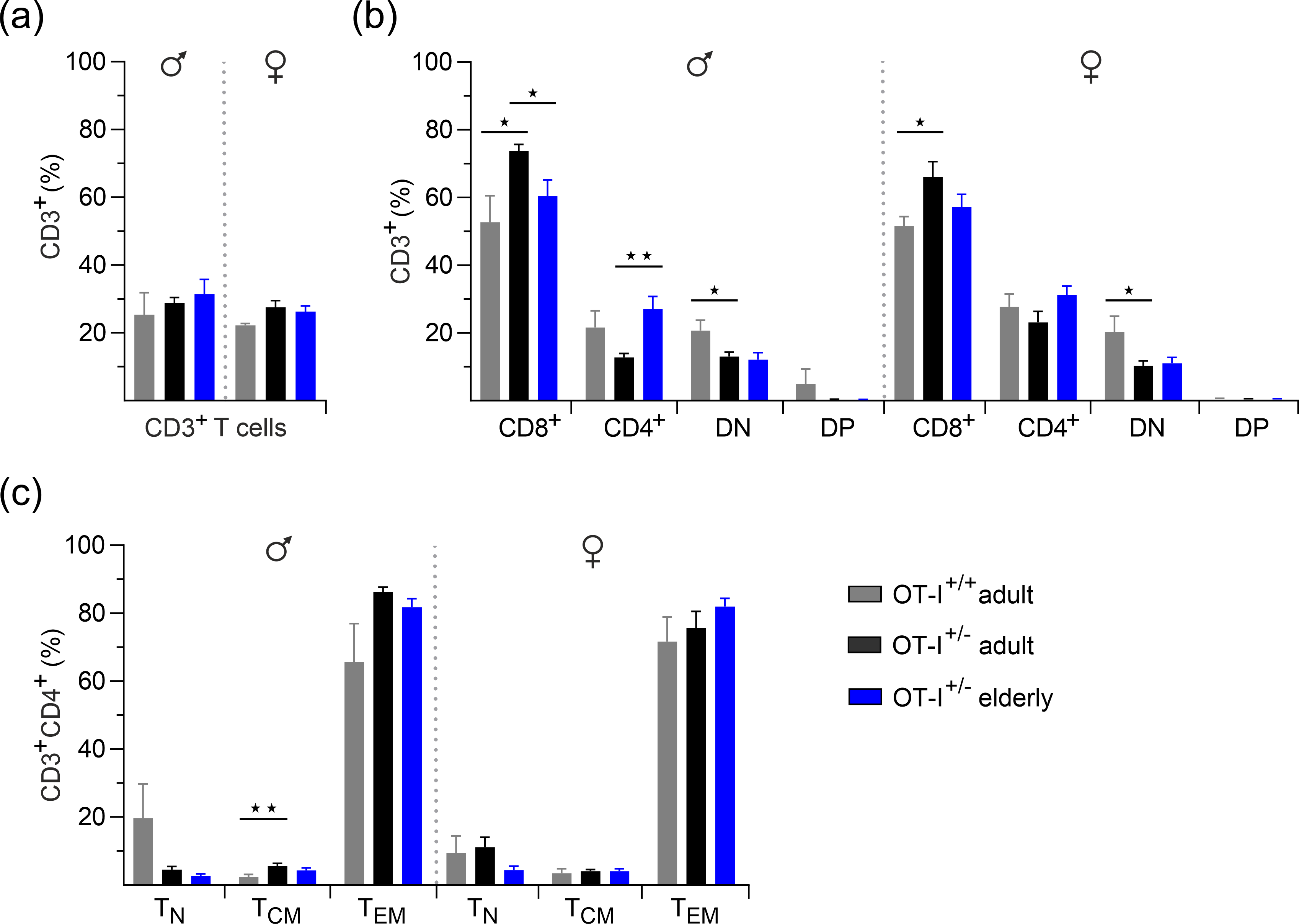


FIGURE S1 Distribution of splenic T cells in OT-I^+/+^ and OT-I^+/-^ mice. Flow cytometry-based analysis of T cell subpopulations in adult and elderly OT-I^+/-^ (n=8-11) compared to OT‑I^+/+^ mice (n=6) in both males and females. (a) Percentage of total CD3^+^ T cells in isolated splenocytes. (b) Percentage of CD8^+^, CD4^+^, double negative (DN) and double positive (DP) proportion within CD3^+^ T cells. (c) Distribution of memory subsets in CD4^+^ T cells. T cell subsets were defined based on CD62L and CD44 surface expression: T_N_: CD62L^high^CD44^low^, T_CM_: CD62L^high^CD44^high^ and T_EM_: CD62L^low^CD44^high^. Data are presented as mean ± SEM.


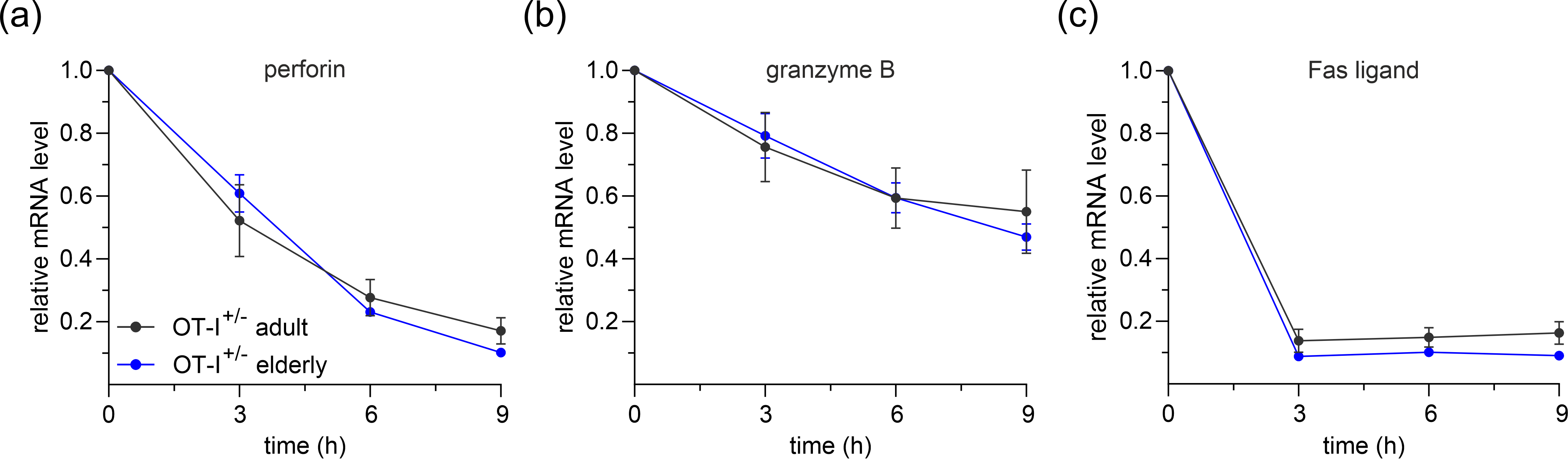


FIGURE S2 CD8^+^ T cells from elderly OT-I^+/-^ mice show similar kinetics of mRNA degradation. Relative mRNA abundance of perforin (a), granzyme B (b), and Fas ligand (c) after addition of transcription inhibitor actinomycin D. Activated OVA-specific CD8^+^ T cells from adult and elderly OT-I^+/-^ mice were incubated with 10 µg/ml actinomycin D and mRNA decay was evaluated by normalizing mRNA levels of each indicated time point to mRNA levels at time point 0. Data are presented as mean ± SEM, n=4.
